# Supplementary material for: Stroke and Athletes: A Scoping Review
Source: Int J Environ Res Public Health. 2021 Sep 24;18(19):10047. doi: 10.3390/ijerph181910047 (PMC8507848; doi:10.3390/ijerph181910047)
Supplement: Supplementary file 1 [file ijerph-18-10047-s001.zip › ijerph-1307006-supplementary.pdf]

Table S1:
Stroke and Athletes
Final Search Strategies

Ovid MEDLINE(R) and Epub Ahead of Print, In-Process & Other Non-Indexed Citations and Daily

#	Searches	Results
1	stroke/ or stroke, lacunar/	100500
2	*brain ischemia/ or *brain infarction/ or *ischemic attack, transient/ or *vertebrobasilar insufficiency/	58434
3	*Cerebrovascular Disorders/ or *Cerebral Infarction/	45929
4	*intracranial hemorrhages/ or *cerebral hemorrhage/ or *subarachnoid hemorrhage/	40714
5	*Vertebral Artery Dissection/ or *Carotid Artery, Internal, Dissection/	2115
6	*Intracranial Embolism/ or *Intracranial Aneurysm/	28293
7	*hemiplegia/ or exp *paresis/	11760
8	(stroke or strokes).ti,kf.	115630
9	((ischemic or ischaemic or lacunar or acute) adj2 stroke*).tw,kf.	66188
10	((cerebrovasc* or cerebral vasc*) adj2 (injur* or disease* or incident* or accident*)).tw,kf.	31129
11	((brain* or cerebr* or cerebell* or intracerebral or intracranial or subarachnoid) adj2 (haemorrhage* or hemorrhage* or haematoma* or hematoma* or bleed*)).tw,kf.	64550
12	((artery or arterial) adj2 dissection).tw,kf.	6338
13	(transient adj2 attack*).tw,kf.	14378
14	(hemipleg* or hemipar* or paresis or paretic or hemineglect or hemi-neglect or ((unilateral or spatial or hemi?spatial or visual) adj2 neglect)).tw,kf.	38880
15	or/1-13	332757
16	exp Athletic Injuries/ or exp Athletes/ or exp Para-Athletes/	38180
17	Sports/ or Sports medicine/	39179
18	exp baseball/ or exp basketball/ or exp Bicycling/ or exp boxing/ or exp football/ or exp gymnastics/ or exp hockey/ or exp martial arts/ or exp racquet sports/ or exp skating/ or exp Skiing/ or exp snow sports/ or exp soccer/ or exp Swimming/ or exp Tennis/ or exp "track and field"/ or exp volleyball/ or exp water sports/ or exp wrestling/ or exp youth sports/	66841
19	(athlete* or athletic* or para-athlete* or parathlet* or Paralympic* or para-olympic*or olympic*).tw,kf.	61654
20	sport*.tw,kf.	77372
21	(baseball or basketball or biking or bicycling* or bmx or boxing or "bull rid*" or bullrid* or cheerleading or "cheer leading" or climbing or cricket or diving or equestrian or football or golf or gymnastics or handball or "horse* riding" or hockey or lacrosse or mountaineering or netball or "net ball" or "racquet sport*" or racquetball or ringette or "roller derb*" or rollerskat* or rodeo* or rugby or skateboard* or skating or skiing or snowboard* or "snow sport*" or soccer or softball or squash or swimming or tennis or "track and field" or wrestling or volleyball).tw,kf.	92372
22	(archery or badminton or bobsled* or bobsleigh* or canoe* or "cross country" or fencing or kayak* or luge or rifle or rowing or sailing or skeleton or "ski jump*" or	49236

	sledding or surfing or trampolin* or "water polo" or "weight lifting" or windsurfing or yachting).tw,kf.	
23	(biker* or boxer* or "cheer leader*" or cheerleader* or climber* or cyclist* or diver or divers or fencer* or fighter* or footballer* or goalie* or golfer* or gymnast or gymnasts or "horse* rider*" or jockey* or judoka* or mountaineer* or rower* or sailor* or skater* or skier* or sledder* or snowboarder* or surfer* or swimmer* or "weight lifter*" or wrestler*).tw,kf.	29223
24	or/16-23	276295
25	15 and 24	2118
26	(stroke* adj2 (volume or heat or length or repetition* or quality or cycle* or asymmetrical or symmetrical or power or distance or index or gearing or kinematic* or velocity or biomechanics or mechanics or technique* or amplitude*)).tw,kf.	24240
27	(Stroke* adj3 (volley or overhand or underhand or overhead or crawl or rally or rallies or forehand or backhand or putting or smash* or backward or forward or special)).tw,kf.	616
28	(Stroke* adj1 (swimming or swim or kayak* or rowing or badminton or volleyball or tennis or golf* or paddl* or racket or racquet or practice or ball or balls or oar* or canoe*)).tw,kf.	309
29	or/26-28	24895
30	25 not 29	1688
31	limit 30 to "humans only (removes records about animals)"	1511
32	(Rat or rats or fish* or duck* or worm* or zebrafish* or mice or mouse or fin or fins or rabbit* or dog or dogs or animal-model* or bovine or rodent* or whale* or lion*).ti.	1775113
33	31 not 32	1487
34	limit 33 to (address or autobiography or bibliography or biography or clinical trial, veterinary or clinical trials, veterinary as topic or dataset or dictionary or directory or interactive tutorial or legal case or legislation or observational study, veterinary or patient education handout or portrait or randomized controlled trial, veterinary or "systematic review" or systematic reviews as topic or video-audio media or webcast)	64
35	33 not 34	1423
36	limit 35 to english language	1253

Embase

#	Searches	Results
1	cerebrovascular accident/ or lacunar stroke/	205337
2	*brain ischemia/ or *transient ischemic attack/	84968
3	*brain infarction/ or *cerebrovascular disease/ or *cerebellum infarction/	41991
4	*brain hemorrhage/ or *cerebellum hemorrhage/ or *subarachnoid hemorrhage/	52440
5	*artery dissection/ or *carotid artery injury/	6682
6	*brain embolism/ or *intracranial aneurysm/	13920
7	*hemiplegia/ or *paresis/	7892
8	(stroke or strokes).ti,kw.	191141
9	((ischemic or ischaemic or lacunar or acute) adj2 stroke*).tw,kw.	115804

10	((cerebrovasc* or cerebral vasc*) adj2 (injur* or disease* or incident* or accident*)).tw,kw.	47662
11	((brain* or cerebr* or cerebell* or intracerebral or intracranial or subarachnoid) adj2 (haemorrhage* or hemorrhage* or haematoma* or hematoma* or bleed*)).tw,kw.	91098
12	((artery or arterial) adj2 dissection).tw,kw.	9339
13	(transient adj2 attack*).tw,kw.	22225
14	(hemipleg* or hemipar* or paresis or paretic or hemineglect or hemi-neglect or ((unilateral or spatial or hemi?spatial or visual) adj2 neglect)).tw,kw.	52728
15	or/1-14	553811
16	athlete/ or basketball player/ or body builder/ or boxer/ or cyclist/ or football player/ or hockey player/ or judoka/ or skier/ or soccer player/ or triathlete/ or wrestler/ or disabled athlete/	52574
17	sport/ or aquatic sport/ or exp athletics/ or baseball/ or basketball/ or body building/ or boxing/ or exp combat sport/ or contact sport/ or "cricket (sport)"/ or cycling/ or diving/ or endurance sport/ or extreme sport/ or football/ or hockey/ or horseback riding/ or ice hockey/ or martial art/ or mountaineering/ or exp racquet sport/ or rock climbing/ or roller skating/ or rowing/ or rugby/ or skateboarding/ or skating/ or skiing/ or soccer/ or swimming/ or team sport/ or exp tennis/ or "track and field"/ or volleyball/ or water skiing/ or winter sport/ or wrestling/ or youth sport/	128220
18	sport injury/	29468
19	sports medicine/	16838
20	(athlete* or athletic* or para-athlete* or parathlet* or Paralympic* or para-olympic*or olympic*).tw,kw.	72995
21	sport*.tw,kw.	102114
22	(baseball or basketball or biking or bicycling* or bmx or boxing or "bull rid*" or bullrid* or cheerleading or "cheer leading" or climbing or cricket or diving or equestrian or football or golf or gymnastics or handball or "horse* riding" or hockey or lacrosse or mountaineering or netball or "net ball" or "racquet sport*" or racquetball or ringette or "roller derb*" or rollerskat* or rodeo* or rugby or skateboard* or skating or skiing or snowboard* or "snow sport*" or soccer or softball or squash or swimming or tennis or "track and field" or wrestling or volleyball).tw,kw.	107745
23	(archery or badminton or bobsled* or bobsleigh* or canoe* or "cross country" or fencing or kayak* or luge or rifle or rowing or sailing or skeleton or "ski jump*" or sledding or surfing or trampolin* or "water polo" or "weight lifting" or windsurfing or yachting).tw,kw.	63011
24	(biker* or boxer* or "cheer leader*" or cheerleader* or climber* or cyclist* or diver or divers or fencer* or fighter* or footballer* or goalie* or golfer* or gymnast or gymnasts or "horse* rider*" or jockey* or judoka* or mountaineer* or rower* or sailor* or skater* or skier* or sledder* or snowboarder* or surfer* or swimmer* or "weight lifter*" or wrestler*).tw,kw.	33025
25	or/16-24	339576
26	15 and 25	4078
27	(stroke* adj2 (volume or heat or length or repetition* or quality or cycle* or asymmetrical or symmetrical or power or distance or index or gearing or kinematic* or velocity or biomechanics or mechanics or technique* or amplitude*)).tw,kw.	35163

28	(Stroke* adj3 (volley or overhand or underhand or overhead or crawl or rally or rallies or forehand or backhand or putting or smash* or backward or forward or special)).tw,kw.	850
29	(Stroke* adj1 (swimming or swim or kayak* or rowing or badminton or volleyball or tennis or golf* or paddl* or racket or racquet or practice or ball or balls or oar* or canoe*)).tw,kw.	408
30	or/27-29	36019
31	26 not 30	3364
32	limit 31 to "humans only (removes records about animals)"	3034
33	(Rat or rats or fish* or duck* or worm* or zebrafish* or mice or mouse or fin or fins or rabbit* or dog or dogs or animal-model* or bovine or rodent* or whale* or lion*).ti.	1894195
34	32 not 33	3010
35	limit 34 to conference abstracts	814
36	34 not 35	2196
37	limit 36 to (book or book series or conference proceeding or major reference work or trade journal)	6
38	36 not 37	2190
39	limit 38 to english language	1883

CINAHL Plus with Full Text

#	Query	Results
S1	(MH "Stroke") OR (MH "Stroke, Lacunar")	72,632
S2	(MM "Cerebrovascular Disorders") OR (MM "Cerebral Ischemia") OR (MM "Cerebral Ischemia, Transient")	18,023
S3	(MM "Intracranial Hemorrhage") OR (MM "Cerebral Hemorrhage") OR (MM "Hematoma, Subdural") OR (MM "Subarachnoid Hemorrhage")	10,829
S4	(MM "Vertebral Artery Dissections") OR (MM "Carotid Artery Dissections") OR (MM "Arterial Dissections") OR (MM "Intracranial Arterial Diseases") OR (MM "Intracranial Embolism and Thrombosis")	2,228
S5	(MM "Hemiplegia")	4,540
S6	TI (stroke or strokes)	56,848
S7	TI (((ischemic or ischaemic or lacunar or acute) N2 stroke*)) OR AB (((ischemic or ischaemic or lacunar or acute) N2 stroke*))	27,080
S8	TI (((cerebrovasc* or cerebral vasc*) N2 (injur* or disease* or incident* or accident*))) OR AB (((cerebrovasc* or cerebral vasc*) N2 (injur* or disease* or incident* or accident*)))	8,277
S9	TI (((brain* or cerebr* or cerebell* or intracerebral or intracranial or subarachnoid) N2 (haemorrhage* or hemorrhage* or haematoma* or hematoma* or bleed*))) OR AB (	15,112

	((brain* or cerebr* or cerebell* or intracerebral or intracranial or subarachnoid) N2 (haemorrhage* or hemorrhage* or haematoma* or hematoma* or bleed*)))	
S10	TI (((artery or arterial) N2 dissection)) OR AB (((artery or arterial) N2 dissection))	2,177
S11	TI (transient N2 attack*) OR AB (transient N2 attack*)	4,950
S12	TI ((hemipleg* or hemipar* or paresis or paretic or hemineglect or hemi-neglect or ((unilateral or spatial or hemi?spatial or visual) N2 neglect))) OR AB ((hemipleg* or hemipar* or paresis or paretic or hemineglect or hemi-neglect or ((unilateral or spatial or hemi?spatial or visual) N2 neglect))(transient N2 attack*))	4,492
S13	S1 OR S2 OR S3 OR S4 OR S5 OR S6 OR S7 OR S8 OR S9 OR S10 OR S11 OR S12	120,484
S14	MH "Sports") OR (MH "Horseback Riding") OR (MH "Animal Sports") OR (MH "Amateur Sports") OR (MH "Aquatic Sports") OR (MH "Diving") OR (MH "Rowing") OR (MH "Swimming") OR (MH "Athletic Performance") OR (MH "Athletic Training") OR (MH "Athletic Training Programs") OR (MH "Sport Specific Training") OR (MH "Body Building") OR (MH "College Sports") OR (MH "Contact Sports") OR (MH "Boxing") OR (MH "Football") OR (MH "Martial Arts") OR (MH "Rugby") OR (MH "Wrestling") OR (MH "Cycling") OR (MH "Endurance Sports") OR (MH "Extreme Sports") OR (MH "Fencing") OR (MH "Golf") OR (MH "Gymnastics") OR (MH "Handball") OR (MH "Mountaineering") OR (MH "Professional Sports") OR (MH "Racquet Sports") OR (MH "Tennis") OR (MH "Rock Climbing") OR (MH "Skating") OR (MH "Ice Skating") OR (MH "Skateboarding") OR (MH "Skiing") OR (MH "Snow Skiing") OR (MH "Sporting Events") OR (MH "Sports, Disabled") OR (MH "Sports Participation") OR (MH "Target Sports") OR (MH "Archery") OR (MH "Baseball") OR (MH "Team Sports") OR (MH "Basketball") OR (MH "Cricket (Sports)") OR (MH "Hockey") OR (MH "Soccer") OR (MH "Softball") OR (MH "Volleyball") OR (MH "Track and Field") OR (MH "Weight Lifting") OR (MH "Winter Sports") OR (MH "Snowboarding") OR (MH "Cross Country Skiing")	74,359
S15	(MH "Athletic Injuries") OR (MH "Aquatic Sports Injuries") OR (MH "Skiing Injuries") OR (MH "Swimming Injuries") OR (MH "Baseball Injuries") OR (MH "Basketball Injuries") OR (MH "Boxing Injuries") OR (MH "Cricket Injuries") OR (MH "Cycling Injuries") OR (MH "Fencing Injuries") OR (MH "Football Injuries") OR (MH "Golf Injuries") OR (MH "Gymnastics Injuries") OR (MH "Hockey Injuries") OR (MH "Martial Arts Injuries") OR (MH "Mountaineering Injuries") OR (MH "Racquet Sports Injuries") OR (MH "Tennis Injuries") OR (MH "Rock Climbing Injuries") OR (MH "Rugby Injuries") OR (MH "Skateboarding Injuries") OR (MH "Soccer Injuries") OR (MH "Volleyball Injuries") OR (MH "Winter Sports Injuries") OR (MH "Snowboarding Injuries")	22,688
S16	(MH "Sports Medicine") or (MH "Athletes+")	35,731
S17	TI sport* OR AB sport*	49,742
S18	TI ((baseball or basketball or biking or bicycling* or bmx or boxing or bullrid* or "bull rid*" or cheerleading or "cheer leading" or climbing or cricket or diving or equestrian or football or golf or gymnastics or handball or "horse* riding" or hockey or lacrosse or mountaineering or netball or “net ball” or “racquet sport*” or racquetball or ringette or rodeo* or "roller derb*" or rollerskat* or rugby or skateboard* or skating or skiing or snowboard* or “snow sport*” or soccer or softball or squash or swimming or tennis or	38,144

	"track and field" or wrestling or volleyball)) OR AB ((baseball or basketball or biking or bicycling* or bmx or boxing or bullrid* or "bull rid*" or cheerleading or "cheer leading" or climbing or cricket or diving or equestrian or football or golf or gymnastics or handball or "horse* riding" or hockey or lacrosse or mountaineering or netball or “net ball” or “racquet sport*” or racquetball or ringette or rodeo* or "roller derb*" or rollerskat* or rugby or skateboard* or skating or skiing or snowboard* or “snow sport*” or soccer or softball or squash or swimming or tennis or "track and field" or wrestling or volleyball))	
S19	TI ((archery or badminton or bobsled* or bobsleigh* or canoe* or "cross country" or fencing or kayak* or luge or rifle or rowing or sailing or skeleton or "ski jump*" or sledding or surfing or trampolin* or "water polo" or "weight lifting" or windsurfing or yachting)) OR AB ((archery or badminton or bobsled* or bobsleigh* or canoe* or "cross country" or fencing or kayak* or luge or rifle or rowing or sailing or skeleton or "ski jump*" or sledding or surfing or trampolin* or "water polo" or "weight lifting" or windsurfing or yachting))	8,804
S20	TI ((aikido or judo or jiujitsu or "jiu-jitsu" or jujitsu or "ju jitsu" or karate or kickbox* or “Martial art*” or taekwondo or “tae kwon do” or “tai ji”)) OR AB ((aikido or judo or jiujitsu or "jiu-jitsu" or jujitsu or "ju jitsu" or karate or kickbox* or “Martial art*” or taekwondo or “tae kwon do” or “tai ji”))	1,546
S21	TI ((athlete* or athletic* or para-athlete* or parathlet* or Paralympic* or para-olympic*or olympic*)) OR AB ((athlete* or athletic* or para-athlete* or parathlet* or Paralympic* or para-olympic*or olympic*))	39,896
S22	TI (((biker* or boxer* or "cheer leader*" or cheerleader* or climber* or cyclist* or diver or divers or fencer* or fighter* or footballer* or goalie* or golfer* or gymnast or gymnasts or "horse* rider*" or jockey* or judoka* or mountaineer* or rower* or sailor* or skater* or skier* or sledder* or snowboarder* or surfer* or swimmer* or "weight lifter*" or wrestler*))) OR AB (((biker* or boxer* or "cheer leader*" or cheerleader* or climber* or cyclist* or diver or divers or fencer* or fighter* or footballer* or goalie* or golfer* or gymnast or gymnasts or "horse* rider*" or jockey* or judoka* or mountaineer* or rower* or sailor* or skater* or skier* or sledder* or snowboarder* or surfer* or swimmer* or "weight lifter*" or wrestler*)))	13,041
S23	S14 OR S15 OR S16 OR S17 OR S18 OR S19 OR S20 OR S21 OR S22	145,804
S24	S13 AND S23	1,166
S25	TI ((stroke* N2 (volume or heat or length or repetition* or quality or cycle* or asymmetrical or symmetrical or power or distance or index or gearing or kinematic* or velocity or biomechanics or mechanics or technique* or amplitude*))) OR AB ((stroke* N2 (volume or heat or length or repetition* or quality or cycle* or asymmetrical or symmetrical or power or distance or index or gearing or kinematic* or velocity or biomechanics or mechanics or technique* or amplitude*)))	6,297
S26	TI ((Stroke* N3 (volley or overhand or underhand or overhead or crawl or rally or rallies or forehand or backhand or putting or smash* or backward or forward or special))) OR AB ((Stroke* N3 (volley or overhand or underhand or overhead or crawl	302

	or rally or rallies or forehand or backhand or putting or smash* or backward or forward or special)))	
S27	TI ((Stroke* N1 (swimming or swim or kayak* or rowing or badminton or volleyball or tennis or golf* or paddl* or racket or racquet or practice or ball or balls or oar* or canoe*))) OR AB ((Stroke* N1 (swimming or swim or kayak* or rowing or badminton or volleyball or tennis or golf* or paddl* or racket or racquet or practice or ball or balls or oar* or canoe*)))	514
S28	S25 OR S26 OR S27	6,924
S29	S24 NOT S28	922
S30	TI (Rat or rats or fish* or duck* or worm* or zebrafish* or mice or mouse or fin or fins or rabbit* or dog or dogs or animal-model* or bovine or rodent* or whale* or lion*)	93,283
S31	S29 NOT S30	903
S32	S29 NOT S30<br>Narrow by Language; English	886

SportDiscus with Full Text

#	Query	Results
S1	DE "STROKE"	1,802
S2	DE "CEREBROVASCULAR disease" OR DE "CEREBRAL embolism & thrombosis" OR DE "CEREBRAL hemorrhage"	2,838
S3	DE "CEREBRAL hemorrhage"	134
S4	DE "CEREBRAL embolism & thrombosis"	45
S5	DE "HEMIPLEGIA" OR DE "PARALYSIS"	1,673
S6	TI (stroke or strokes)	8,287
S7	TI ((((ischemic or ischaemic or lacunar or acute) N2 stroke*))) OR AB ((((ischemic or ischaemic or lacunar or acute) N2 stroke*))) OR KW ((((ischemic or ischaemic or lacunar or acute) N2 stroke*)))	1,233
S8	TI ((((cerebrovasc* or cerebral vasc*) N2 (injur* or disease* or incident* or accident*)))) OR AB ((((cerebrovasc* or cerebral vasc*) N2 (injur* or disease* or incident* or accident*)))) OR KW ((((cerebrovasc* or cerebral vasc*) N2 (injur* or disease* or incident* or accident*))))	772
S9	TI ((((brain* or cerebr* or cerebell* or intracerebral or intracranial or subarachnoid) N2 (haemorrhage* or hemorrhage* or haematoma* or hematoma* or bleed*)))) OR AB ((((brain* or cerebr* or cerebell* or intracerebral or intracranial or subarachnoid) N2 (haemorrhage* or hemorrhage* or haematoma* or hematoma* or bleed*)))) OR KW ((((brain* or cerebr* or cerebell* or intracerebral or intracranial or	513

	subarachnoid) N2 (haemorrhage* or hemorrhage* or haematoma* or hematoma* or bleed*))))	
S10	TI ((((artery or arterial) N2 dissection))) OR AB ((((artery or arterial) N2 dissection))) OR KW ((((artery or arterial) N2 dissection)))	117
S11	TI (transient N2 attack*) OR AB (transient N2 attack*) OR KW (transient N2 attack*)	175
S12	TI (((hemipleg* or hemipar* or paresis or paretic or hemineglect or hemi-neglect or ((unilateral or spatial or hemi?spatial or visual) N2 neglect)))) OR AB (((hemipleg* or hemipar* or paresis or paretic or hemineglect or hemi-neglect or ((unilateral or spatial or hemi?spatial or visual) N2 neglect)))) OR KW (((hemipleg* or hemipar* or paresis or paretic or hemineglect or hemi-neglect or ((unilateral or spatial or hemi?spatial or visual) N2 neglect))))	2,796
S13	S1 OR S2 OR S3 OR S4 OR S5 OR S6 OR S7 OR S8 OR S9 OR S10 OR S11 OR S12	13,474
S14	DE "SPORTS" OR DE "AMATEUR sports" OR DE "AQUATIC sports" OR DE "BALL games" OR DE "COLLEGE sports" OR DE "CONTACT sports" OR DE "ENDURANCE sports" OR DE "EXTREME sports" OR DE "INDIVIDUAL sports" OR DE "MILITARY sports" OR DE "OLYMPIC Games" OR DE "PROFESSIONAL sports" OR DE "RECREATIONAL sports" OR DE "SCHOOL sports" OR DE "SPORTS competitions" OR DE "TEAM sports" OR DE "VIOLENCE in sports" OR DE "WINTER sports" OR DE "SPORTS camps" OR DE "SPORTS events" OR DE "SPORTS medicine" OR DE "SPORTS participation"	209,876
S15	DE "ATHLETICS" OR DE "BASEBALL" OR DE "BASKETBALL" OR DE "BICYCLE motocross" OR DE "BICYCLE racing" OR DE "BICYCLE racing training" OR DE "BOXING" OR DE "CYCLING" OR DE "CHEERLEADING" OR DE "ROCK climbing" OR DE "CRACK climbing" OR DE "FACE climbing" OR DE "FREE climbing" OR DE "INDOOR rock climbing" OR DE "CRICKET (Sport)" OR DE "CRICKET competitions" OR DE "INDOOR cricket" OR DE "TWENTY20 cricket" OR DE "ACROBATIC diving" OR DE "SPRINGBOARD diving" OR DE "HORSE sports" OR DE "EQUESTRIAN accidents" OR DE "DRESSAGE tests" OR DE "EVENTING (Horsemanship)" OR DE "GAMES on horseback" OR DE "HORSE racing" OR DE "SHOW jumping" OR DE "SHOW riding" OR DE "VAULTING (Horsemanship)" OR DE "FOOTBALL" OR DE "CANADIAN football" OR DE "COLLEGE football" OR DE "HIGH school football" OR DE "MINOR league football" OR DE "PROFESSIONAL football" OR DE "ARENA football" OR DE "RUGBY football" OR DE "COLLEGE rugby football" OR DE "FLORENTINE football" OR DE "PROFESSIONAL rugby football" OR DE "RUGBY League football" OR DE "RUGBY Union football" OR DE "RUGBY competitions" OR DE "SEVEN-a-side rugby football" OR DE "AUSTRALIAN football" OR DE "TACKLING (Rugby)" OR DE "GOLF" OR DE "COLLEGE golf" OR DE "PROFESSIONAL golf" OR DE "SNOW golf" OR DE "GYMNASTICS" OR DE "ARTISTIC gymnastics" OR DE "SWEDISH gymnastics" OR DE "TEAM aerobics" OR DE "TUMBLING" OR DE "HANDBALL" OR DE "TEAM handball" OR DE "HOCKEY" OR DE "COLLEGE hockey" OR DE "FIELD hockey" OR DE "INDOOR hockey" OR DE "LAWN hockey" OR DE "MINOR league hockey" OR DE "ROLLER hockey" OR DE "BALL hockey" OR DE "PROFESSIONAL hockey" OR DE "LACROSSE" OR DE "MOUNTAINEERING" OR DE "NETBALL" OR DE "RACKET games" OR DE "RACQUETBALL" OR DE "RINGETTE (Game)" OR DE "RODEOS" OR DE "BRONC riding" OR DE "BULL riding" OR DE "CALF roping" OR DE "CHUCKWAGON racing" OR DE "STEER roping" OR DE "STEER wrestling" OR DE "TEAM penning" OR DE "TEAM	413,378

	roping" OR DE "TRICK roping" OR DE "SKATEBOARDING" OR DE "SNOW skating" OR DE "SKATING" OR DE "FIGURE skating" OR DE "FREE skating" OR DE "ICE dancing" OR DE "PROFESSIONAL skating" OR DE "SPEED skating" OR DE "ROLLER skating" OR DE "SKI acrobatics" OR DE "SKI cross" OR DE "SKI mountaineering" OR DE "SKI racing" OR DE "SKIS & skiing" OR DE "SLALOM canoeing" OR DE "SLALOM racing" OR DE "SLALOM skiing" OR DE "SNOWBOARDING" OR DE "BACKCOUNTRY snowboarding" OR DE "SLOPESTYLE snowboarding" OR DE "SKIBOARDING" OR DE "SOCCER" OR DE "COLLEGE soccer" OR DE "HIGH school soccer" OR DE "INDOOR soccer" OR DE "PROFESSIONAL soccer" OR DE "SOFTBALL" OR DE "SLOW pitch softball" OR DE "YOUTH league softball" OR DE "SQUASH (Game)" OR DE "SWIMMING" OR DE "SWIMMING competitions" OR DE "SYNCHRONIZED swimming" OR DE "TENNIS" OR DE "COLLEGE tennis" OR DE "COURT tennis" OR DE "PROFESSIONAL tennis" OR DE "TABLE tennis" OR DE "TENNIS leagues" OR DE "TENNIS tournaments" OR DE "TENNIS training" OR DE "TRACK & field" OR DE "ALL-around (Track & field)" OR DE "COLLEGE track & field" OR DE "HURDLING (Track & field)" OR DE "INDOOR track & field" OR DE "JUMPING" OR DE "STEEPLECHASING (Track & field)" OR DE "VAULTING" OR DE "VOLLEYBALL" OR DE "BEACH volleyball" OR DE "COLLEGE volleyball" OR DE "PROFESSIONAL volleyball" OR DE "VOLLEYBALL competitions" OR DE "WRESTLING" OR DE "COLLEGE wrestling" OR DE "FREESTYLE wrestling" OR DE "HIGH school wrestling" OR DE "PROFESSIONAL wrestling" OR DE "SAMBO wrestling" OR DE "SUMO"	
S16	DE "AIKIDO" OR DE "JUDO" OR DE "SAN-jitsu" OR DE "JIU-jitsu" OR DE "KARATE" OR DE "TAE kwon do" OR DE "KICKBOXING" OR DE "MARTIAL arts" OR DE "CAPOEIRA (Dance)" OR DE "MIXED martial arts" OR DE "ARCHERY" OR DE "BADMINTON (Game)" OR DE "BADMINTON tournaments" OR DE "BADMINTON training" OR DE "CANOE racing" OR DE "CROSS-country ski racing" OR DE "NORDIC combined" OR DE "SPORT fencing" OR DE "FENCING" OR DE "KAYAKING" OR DE "KAYAKING training" OR DE "ICE luge racing" OR DE "SHOOTING contests" OR DE "ROWING" OR DE "SAILING" OR DE "SKELETON" OR DE "SKI jumping" OR DE "SLEDDING" OR DE "SURFING" OR DE "BODYBOARDING" OR DE "KITE surfing" OR DE "STAND-up paddle surfing" OR DE "WINDSURFING" OR DE "TRAMPOLINING" OR DE "WATER polo" OR DE "WEIGHT lifting" OR DE "YACHTING"	58,285
S17	DE "ATHLETES" OR DE "ATHLETES' health" OR DE "ARCHERS" OR DE "BADMINTON players" OR DE "BASEBALL players" OR DE "BASKETBALL players" OR DE "BOBSLEDDERS" OR DE "BODYBUILDERS" OR DE "BOXERS (Sports)" OR DE "BULLFIGHTERS" OR DE "CANOEISTS" OR DE "COLLEGE athletes" OR DE "CRICKET players" OR DE "CYCLISTS" OR DE "DIVERS" OR DE "ELITE athletes" OR DE "ENDURANCE athletes" OR DE "FENCERS" OR DE "FOOTBALL players" OR DE "GOLFERS" OR DE "GYMNASTS" OR DE "HANDBALL players" OR DE "HIGH school athletes" OR DE "HOCKEY players" OR DE "JUNIOR high school athletes" OR DE "LACROSSE players" OR DE "MALE athletes" OR DE "MARTIAL artists" OR DE "MIDDLE school athletes" OR DE "MOUNTAINEERS" OR DE "NETBALL players" OR DE "OFFENSIVE players" OR DE "OLDER athletes" OR DE "OLYMPIC athletes" OR DE "PROFESSIONAL athletes" OR DE "ROWERS" OR DE "RUGBY football players" OR DE "RUGBY football teams" OR DE "SKATEBOARDERS" OR DE "SKATERS" OR DE "SKIERS" OR DE "SNOWBOARDERS" OR DE "SOCCER players" OR DE "SOFTBALL players" OR DE	283,712

	"SQUASH players" OR DE "SURFERS" OR DE "SWIMMERS" OR DE "TABLE tennis players" OR DE "TEAM handball players" OR DE "TENNIS players" OR DE "TRACK & field athletes" OR DE "TRIATHLETES" OR DE "VOLLEYBALL players" OR DE "WATER polo players" OR DE "WEIGHT lifters" OR DE "WINDSURFERS (Persons)" OR DE "WOMEN athletes" OR DE "WRESTLERS"	
S18	DE "MARTIAL artists" OR DE "ARCHERS" OR DE "BADMINTON players" OR DE "CANOEISTS" OR DE "KAYAKERS" OR DE "SAILORS" OR DE "SURFERS" OR DE "TRAMPOLINISTS" OR DE "WATER polo players" OR DE "WEIGHT lifters"	6,801
S19	DE "SPORTS medicine" OR DE "PEDIATRIC sports medicine" OR DE "SPORTS emergencies" OR DE "SPORTS nutrition" OR DE "SPORTS ophthalmology" OR DE "SPORTS physical therapy" OR DE "SPORTS injuries" OR DE "AQUATIC sports injuries" OR DE "BASEBALL injuries" OR DE "BASKETBALL injuries" OR DE "BOXING injuries" OR DE "CRICKET injuries" OR DE "DIVING injuries" OR DE "DIVING accidents" OR DE "EQUESTRIAN accidents" OR DE "FOOTBALL injuries" OR DE "GOLF injuries" OR DE "GYMNASTICS injuries" OR DE "HOCKEY injuries" OR DE "HORSE sports injuries" OR DE "IN-line skating injuries" OR DE "JUDO injuries" OR DE "KARATE injuries" OR DE "MARTIAL arts injuries" OR DE "MOTORSPORTS injuries" OR DE "NETBALL injuries" OR DE "RACKET game injuries" OR DE "RUGBY football injuries" OR DE "SKATEBOARDING injuries" OR DE "SKIING injuries" OR DE "SKIING accidents" OR DE "SOCCER injuries" OR DE "TENNIS injuries" OR DE "VAULTING injuries" OR DE "VOLLEYBALL injuries" OR DE "WINTER sports injuries" OR DE "JUDO injuries" OR DE "KARATE injuries" OR DE "MARTIAL arts injuries" OR DE "CANOEING accidents" OR DE "YACHTING accidents"	32,442
S20	TI sport* OR AB sport* OR KW sport*	335,881
S21	TI ((baseball or basketball or biking or bicycling* or bmx or boxing or "bull rid*" or bullrid* or cheerleading or "cheer leading" or climbing or cricket or diving or equestrian or football or golf or gymnastics or handball or "horse* riding" or hockey or lacrosse or mountaineering or netball or “net ball” or “racquet sport*” or racquetball or ringette or rodeo* or "roller derb*" or rollerskat* rugby or skateboard* or skating or skiing or snowboard* or “snow sport*” or soccer or softball or squash or swimming or tennis or "track and field" or wrestling or volleyball)) OR AB ((baseball or basketball or biking or bicycling* or bmx or boxing or "bull rid*" or bullrid* or cheerleading or "cheer leading" or climbing or cricket or diving or equestrian or football or golf or gymnastics or handball or "horse* riding" or hockey or lacrosse or mountaineering or netball or “net ball” or “racquet sport*” or racquetball or ringette or rodeo* or "roller derb*" or rollerskat* rugby or skateboard* or skating or skiing or snowboard* or “snow sport*” or soccer or softball or squash or swimming or tennis or "track and field" or wrestling or volleyball)) OR KW ((baseball or basketball or biking or bicycling* or bmx or boxing or "bull rid*" or bullrid* or cheerleading or "cheer leading" or climbing or cricket or diving or equestrian or football or golf or gymnastics or handball or "horse* riding" or hockey or lacrosse or mountaineering or netball or “net ball” or “racquet sport*” or racquetball or ringette or rodeo* or "roller derb*" or rollerskat* rugby or skateboard* or skating or skiing or snowboard* or “snow sport*” or soccer or softball or squash or swimming or tennis or "track and field" or wrestling or volleyball))	558,146

S22	TI ((archery or badminton or bobsled* or bobsleigh* or canoe* or "cross country" or fencing or kayak* or luge or rifle or rowing or sailing or skeleton or "ski jump*" or sledding or surfing or trampolin* or "water polo" or "weight lifting" or windsurfing or yachting)) OR AB ((archery or badminton or bobsled* or bobsleigh* or canoe* or "cross country" or fencing or kayak* or luge or rifle or rowing or sailing or skeleton or "ski jump*" or sledding or surfing or trampolin* or "water polo" or "weight lifting" or windsurfing or yachting)) OR KW ((archery or badminton or bobsled* or bobsleigh* or canoe* or "cross country" or fencing or kayak* or luge or rifle or rowing or sailing or skeleton or "ski jump*" or sledding or surfing or trampolin* or "water polo" or "weight lifting" or windsurfing or yachting))	52,364
S23	TI ((aikido or judo or jiujitsu or "jiu-jitsu" or jujitsu or "ju jitsu" or karate or kickbox* or “Martial art*” or taekwondo or “tae kwon do” or “tai ji”)) OR AB ((aikido or judo or jiujitsu or "jiu-jitsu" or jujitsu or "ju jitsu" or karate or kickbox* or “Martial art*” or taekwondo or “tae kwon do” or “tai ji”)) OR KW ((aikido or judo or jiujitsu or "jiu-jitsu" or jujitsu or "ju jitsu" or karate or kickbox* or “Martial art*” or taekwondo or “tae kwon do” or “tai ji”))	13,959
S24	TI ((capoeira or "Dim mak" or kenpo or "kung fu" or "pencak silat" or pitfight* or savate or "submission fight*" or UFC or "ultimate fighting champion*")) OR AB ((capoeira or "Dim mak" or kenpo or "kung fu" or "pencak silat" or pitfight* or savate or "submission fight*" or UFC or "ultimate fighting champion*")) OR KW ((capoeira or "Dim mak" or kenpo or "kung fu" or "pencak silat" or pitfight* or savate or "submission fight*" or UFC or "ultimate fighting champion*"))	1,644
S25	TI ((biker* or boxer* or "cheer leader*" or cheerleader* or climber* or cyclist* or diver or divers or fencer* or fighter* or footballer* or goalie* or golfer* or gymnast or gymnasts or "horse* rider*" or jockey* or judoka* or mountaineer* or rower* or sailor* or skater* or skier* or sledder* or snowboarder* or surfer* or swimmer* or "weight lifter*" or wrestler*)) OR AB ((biker* or boxer* or "cheer leader*" or cheerleader* or climber* or cyclist* or diver or divers or fencer* or fighter* or footballer* or goalie* or golfer* or gymnast or gymnasts or "horse* rider*" or jockey* or judoka* or mountaineer* or rower* or sailor* or skater* or skier* or sledder* or snowboarder* or surfer* or swimmer* or "weight lifter*" or wrestler*)) OR KW ((biker* or boxer* or "cheer leader*" or cheerleader* or climber* or cyclist* or diver or divers or fencer* or fighter* or footballer* or goalie* or golfer* or gymnast or gymnasts or "horse* rider*" or jockey* or judoka* or mountaineer* or rower* or sailor* or skater* or skier* or sledder* or snowboarder* or surfer* or swimmer* or "weight lifter*" or wrestler*))	145,733
S26	S14 OR S15 OR S16 OR S17 OR S18 OR S19 OR S20 OR S21 OR S22 OR S23 OR S24 OR S25	1,191,404
S27	S13 AND S26	2,891
S28	TI (((stroke* N2 (volume or heat or length or repetition* or quality or cycle* or asymmetrical or symmetrical or power or distance or index or gearing or kinematic* or velocity or biomechanics or mechanics or technique* or amplitude*)))) OR AB (((stroke* N2 (volume or heat or length or repetition* or quality or cycle* or asymmetrical or symmetrical or power or distance or index or gearing or kinematic*	3,612

	or velocity or biomechanics or mechanics or technique* or amplitude*)))) OR KW (((stroke* N2 (volume or heat or length or repetition* or quality or cycle* or asymmetrical or symmetrical or power or distance or index or gearing or kinematic* or velocity or biomechanics or mechanics or technique* or amplitude*))))	
S29	TI (((Stroke* N3 (volley or overhand or underhand or overhead or crawl or rally or rallies or forehand or backhand or putting or smash* or backward or forward or special)))) OR AB (((Stroke* N3 (volley or overhand or underhand or overhead or crawl or rally or rallies or forehand or backhand or putting or smash* or backward or forward or special)))) OR KW (((Stroke* N3 (volley or overhand or underhand or overhead or crawl or rally or rallies or forehand or backhand or putting or smash* or backward or forward or special))))	960
S30	TI (((Stroke* N1 (swimming or swim or kayak* or rowing or badminton or volleyball or tennis or golf* or paddl* or racket or racquet or practice or ball or balls or oar* or canoe*)))) OR AB (((Stroke* N1 (swimming or swim or kayak* or rowing or badminton or volleyball or tennis or golf* or paddl* or racket or racquet or practice or ball or balls or oar* or canoe*)))) OR KW (((Stroke* N1 (swimming or swim or kayak* or rowing or badminton or volleyball or tennis or golf* or paddl* or racket or racquet or practice or ball or balls or oar* or canoe*))))	1,234
S31	DE "CRAWL stroke (Swimming)" OR DE "BUTTERFLY stroke (Swimming)" OR DE "STROKE volume (Cardiac output)" OR DE "HEAT stroke" OR DE "BACKSTROKE (Swimming)" OR DE "SIDESTROKE (Swimming)" OR DE "ROWING techniques" OR DE "RELEASE (Rowing)" OR DE "BREASTSTROKE (Swimming)"	1,959
S32	S28 OR S29 OR S30 OR S31	6,689
S33	S27 NOT S32	1,772
S34	TI Rat or rats or fish* or duck* or worm* or zebrafish* or mice or mouse or fin or fins or rabbit* or dog or dogs or animal-model* or bovine or rodent* or whale* or lion*	29,683
S35	S33 NOT S34	1,766
S36	S33 NOT S34<br>Narrow by Language: - english<br><br>Excluded books, web-based, dissertations	1,441

Scopus

((((TITLE ((stroke OR strokes)) OR TITLE-ABS-KEY (((ischemic OR ischaemic OR lacunar OR acute) W/2 stroke*)))) OR (TITLE-ABS-KEY (((cerebrovasc* OR "cerebral vasc*") W/2 (injur* OR disease* OR incident* OR accident*)))) OR (TITLE-ABS-KEY (((brain* OR cerebr* OR cerebell* OR intracerebral OR intracranial OR subarachnoid) W/2 (haemorrhage* OR hemorrhage* OR haematoma* OR hematoma* OR bleed*)))) OR ((TITLE-ABS-KEY (((artery OR arterial) W/2 dissection)) OR TITLE-ABS-KEY ((transient W/2 attack*)) OR TITLE-ABS-KEY ((hemipleg* OR hemipar* OR paresis OR paretic OR hemineglect OR hemi-neglect OR ((unilateral OR spatial OR hemi?spatial OR visual) W/2 neglect)))))) AND (TITLE-ABS-KEY (athlete* OR athletic* OR para-athlete* OR parathlet* OR paralympic* OR para-olympic*or AND olympic*) OR ((TITLE-ABS-KEY (baseball OR basketball OR biking OR bicycling* OR bmx OR boxing OR "bull rid*" OR bullrid* OR cheerleading OR "cheer leading" OR climbing OR cricket OR diving OR equestrian OR football OR golf OR gymnastics OR handball OR "horse* riding" OR hockey OR lacro) OR TITLE-ABS-KEY ("horse* riding" OR hockey OR lacrosse OR mountaineering OR netball OR "net ball" OR "racquet sport*" OR racquetball OR ringette OR "roller derb*" OR rollerskat* OR rodeo*) OR TITLE-ABS-KEY (rugby OR skateboard* OR skating OR skiing OR snowboard* OR "snow sport*" OR soccer OR softball OR squash OR swimming OR tennis OR "track and field" OR wrestling OR volleyball) OR TITLE-ABS-KEY (archery OR badminton OR bobsled* OR bobsleigh* OR canoe* OR "cross country" OR fencing OR kayak* OR luge OR rifle) OR TITLE-ABS-KEY (rowing OR sailing OR skeleton OR "ski jump*" OR sledding OR surfing OR trampolin* OR "water polo" OR "weight lifting" OR windsurfing OR yachting))) OR ((TITLE-ABS-KEY (aikido OR judo OR jiujitsu OR "jiu-jitsu" OR jujitsu OR "ju jitsu" OR karate OR kickbox* OR "Martial art*" OR taekwondo OR "tae kwon do" OR "tai ji") OR TITLE-ABS-KEY (capoeira OR "Dim mak" OR kenpo OR "kung fu" OR "pencak silat" OR pitfight* OR savate OR "submission fight*" OR ufc OR "ultimate fighting champion*") OR TITLE-ABS-KEY (biker* OR boxer* OR "cheer leader*" OR cheerleader* OR climber* OR cyclist* OR diver OR divers OR fencer* OR fighter* OR footballer* OR goalie*) OR TITLE-ABS-KEY (golfer* OR gymnast OR gymnasts OR "horse* rider*" OR jockey* OR judoka* OR mountaineer* OR rower* OR sailor* OR skater* OR skier* OR sledder* OR snowboarder* OR surfer* OR swimmer* OR "weight lifter*" OR wrestler*))))) AND NOT ((TITLE-ABS-KEY ((((stroke* W/2 (volume OR heat OR length OR repetition* OR quality OR cycle* OR asymmetrical OR symmetrical OR power OR distance OR index OR gearing OR kinematic* OR velocity OR biomechanics OR mechanics OR technique* OR amplitude*))))) OR TITLE-ABS-KEY ((((stroke* W/3 (volley OR overhand OR underhand OR overhead OR crawl OR rally OR rallies OR forehand OR backhand OR putting OR smash* OR backward OR forward OR special))))) OR TITLE-ABS-KEY ((((stroke* W/1 (swimming OR swim OR kayak* OR rowing OR badminton OR volleyball OR tennis OR golf* OR paddl* OR racket OR racquet OR practice OR ball OR balls OR oar* OR canoe*)))))))) AND NOT INDEX (medline) AND (LIMIT-TO (LANGUAGE , "English")) View less
